# Cord Blood Adipokines and Lipids and Adolescent Nonalcoholic Fatty Liver Disease

**DOI:** 10.1210/jc.2016-2604

**Published:** 2016-09-20

**Authors:** Joy Simpson, Andrew D. Smith, Abigail Fraser, Naveed Sattar, Mark Callaway, Robert S. Lindsay, Debbie A. Lawlor, Scott M. Nelson

**Affiliations:** School of Medicine (J.S., S.M.N.), University of Glasgow, Glasgow G31 2ER, United Kingdom; Medical Research Council Integrative Epidemiology Unit (A.D.S., A.F., D.A.L.), School of Social and Community Medicine (A.D.S., A.F., D.A.L.), University of Bristol, Bristol BS13NY United Kingdom; Institute of Cardiovascular and Metabolic Medicine (N.S., R.S.L.), British Heart Foundation Glasgow Cardiovascular Research Centre, Glasgow G61 1QH, United Kingdom; and University Hospitals Bristol National Health Service Foundation Trust (M.C.), Bristol BS1 3NY, United Kingdom

## Abstract

**Context:**

Maternal adiposity in pregnancy is associated with offspring adiposity and metabolic dysfunction postnatally, including greater risk of nonalcoholic fatty liver disease (NAFLD). Recent genetic analyses suggest a causal effect of greater maternal body mass index on offspring birth weight and ponderal index, but the relative roles of the environment in utero or later in life remains unclear.

**Objective:**

We sought to determine whether markers of infant adiposity (birth weight, umbilical cord blood leptin, adiponectin, and lipids) were associated with markers of NAFLD in adolescence.

**Design, Setting, and Participants:**

This was a UK prospective birth cohort with 17 years of follow-up with liver function tests (aspartate aminotransferase, alanine aminotransferase, gamma-glutamyltransferase) (n = 1037 participants), and ultrasound scan assessed liver fat, volume, and sheer velocity at age 17 (n = 541 participants). Missing covariate data were imputed.

**Main Outcomes:**

Ultrasound and biochemical measures of NAFLD were measured.

**Results:**

Birth weight, cord blood leptin, and adiponectin were not associated with a diagnosis of NAFLD. In adjusted analyses, 2 of 42 associations attained conventional 5% levels of significance. Birth weight was positively associated with liver volume (1.0% greater per 100 g [95% confidence interval 0.5%–2.0%]). Cord high-density lipoprotein-cholesterol was positively associated with alanine aminotransferase (11.6% higher per 1 mmol/L [95% confidence interval 0.3, 23.4]); however, this association was primarily mediated via offspring adiposity.

**Conclusions:**

In this extensive analysis, we found little evidence measurements of infant fat mass and birth size were related to adolescent markers of NAFLD. The association between birth weight and adolescent liver volume may indicate the contribution of greater organ size to birth weight and tracking of organ size.

## Abstract (precis)

In a birth cohort of 1,037 children we found that there was no consistent evidence that birthweight, neonatal adiposity or umbilical cord lipids were associated with liver dysfunction in adolescence.

Nonalcoholic fatty liver disease (NAFLD) is one of the most common causes of chronic liver disease children in the developed world (1). The identification of NAFLD in children and adolescents led to the speculation that intrauterine events may contribute to its early pathogenesis (2, 3). Intrauterine growth restriction and low birth weight have been associated with liver cirrhosis-related mortality (4) and markers of liver damage and function in adults (5). Although the causal effect of the supply of excess nutrients to the fetus, as observed in maternal obesity and diabetes, on increasing birth weight and neonatal adiposity is clear (6, 7), the influence on long-term liver function is less well defined. Mouse and primate models of a maternal high-fat diet during pregnancy exhibit offspring obesity and hepatic lipid accumulation (8, 9). Increasing maternal body mass index (BMI) has also been associated with increased neonatal liver fat deposition (10). Long-term human studies are, however, less consistent with birth weight positively associated with adverse liver function or a diagnosis of NAFLD at age 17 years in some (11) but not all studies (12).

These differences may reflect that assessments of the intrauterine contribution to the onset of metabolic disease in the offspring have primarily used birth weight, which reflects both fetal fat and lean mass. Umbilical cord blood leptin is now widely recognized as an accurate biomarker for neonatal fat mass, with higher levels associated with greater fat mass (13). Cord blood adiponectin is also positively associated with birth weight (14). In mouse studies, overexpression of fetal adiponectin increased the size of fat depots in early life, whereas adiponectin knockout fetuses display lower body weight and lower fat content (15). In the current study, we sought to examine the relationship of neonatal fat mass with blood and ultrasound markers of liver health in adolescents. We used cord blood measurements of leptin and adiponectin as markers of neonatal fat and also examined cord blood lipids with adolescent liver outcomes. To make this a comprehensive assessment, we additionally examined birth weight with later outcomes. In comparison with earlier studies, we were better able to adjust for potential confounding by maternal characteristics including maternal pregnancy BMI, complications of pregnancy, and smoking during pregnancy.

## Design and Methods

### Study population

The Avon Longitudinal Study of Parents and Children (ALSPAC) is a prospective, population-based birth cohort study (16, 17). Full details of the study have been published previously, with the study web site containing details of all the data that are available through a fully searchable data dictionary (http://www.bris.ac.uk/alspac/researchers/data-access/data-dictionary/). Ethical approval was obtained from the ALSPAC Law and Ethics Committee and from the National Health Service local ethics committee.

**Figure 1.**
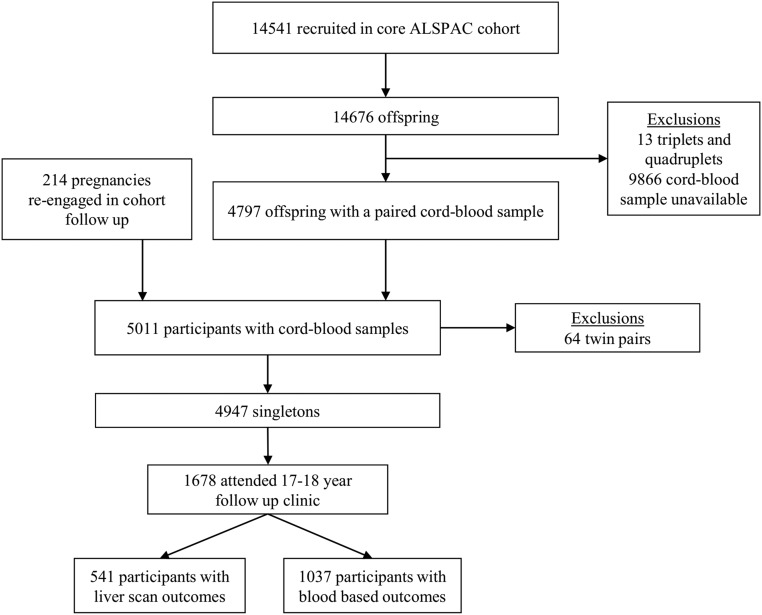
Cohort flow for this analysis.

A total of 14 541 women were initially enrolled and cord blood samples were available for 5011 mother-offspring pairs. Details of all participants who attended the 17–18 follow-up clinic visits (n = 5081) provided blood-based indicators of liver function (n = 3188) and participated in the liver ultrasound substudy (USS) (n = 1887) have previously been reported in detail (18, 19). A detailed outline of the exclusion criteria for this analysis is given in Figure 1, but notably we included participants in the current study if 1) they had a cord blood sample and 2) they attended the 17- to 18-year follow-up study and had blood-based indicators of liver function (n = 1037) or participated in the substudy with liver ultrasound measurement (n = 541).

### Cord blood assays

Cord blood samples were obtained at the time of delivery and spun in preparation for storage at −80°C since then. All cord samples were assayed at the University of Glasgow. Cord leptin and adiponectin were measured using commercially available ELISA kits (quantikine human leptin immunoassay; R&D Systems). Cord lipids (cholesterol, triglycerides, high density lipoproteins (HDL-c) were measured using a Roche Cobas C311 autoanalyzer using enzymatic reagents for lipid determination. Non-high-density lipoprotein (Non-HDL-c) was calculated as total cholesterol minus HDL-c. Analysis of the cord blood was completed within a maximum of three freeze-thaw cycles and remained at −80°C in between. The interassay coefficients of variability were 9.5% for leptin, 3.2% for adiponectin, 4.0% for HDL-c, 3.0% for cholesterol, and 2.7% for triglycerides.

### Assessment of liver outcomes

The protocol for USS assessment in ALSPAC has been published previously (18). Briefly, upper abdominal USS was completed by one of four trained sonographers using a Siemens Acuson S2000 USS system. Echogenicity, our marker of liver fat, was assessed during deep inspiration and recorded as present, absent or uncertain according to established protocols (20). This protocol has high levels of sensitivity and specificity for moderate-severe NAFLD (21, 22). A longitudinal image, in the sagittal plane, with juxtaposition of the right lobe of the liver and the right kidney, was viewed and echogenicity determined by comparison between the liver and kidney. Levels of agreement in identifying echogenicity between the four sonographers was 98% or greater, both immediately after training and at 6-month intervals throughout data collection. Acoustic radiation force impulse imaging of the right lobe of the liver was used to measure liver stiffness (our main indicator of liver stiffness), using standard protocols. Acoustic radiation force impulse imaging, measured as shear velocity in meters per second, was assessed six times with a gap of at least 1 minute between each measurement. The highest and lowest of these measurements were excluded, and the Siemens Acuson S2000 system produced a mean of the remaining four measurements. If this mean was greater than 4 m/sec, a further six measurements were taken from the left lobe. When both right and left lobe values were available, the lowest mean of the two has been used in analyses.

Fasting blood samples (minimum of 6 h) were immediately spun and frozen at −80°C. Measurements were assayed shortly (3–9 mo) after samples were taken with no previous freeze-thaw cycles. All assays were completed in the same laboratory at the University of Glasgow. alanine aminotransferase (ALT), gamma-glutamyltransferase (GGT), and aspartate aminotransferase (AST) were measured by automated analyzer with enzymatic methods. All inter- and intraassay coefficients of variation for these blood-based assays were less than 5%.

### Assessment of covariates

Potential confounders included in this analysis are as follows: maternal age; prepregnancy BMI; smoking status (categorized as never smoked, smoked before but not during pregnancy, and smoked during pregnancy); parity; occupational social class; complications during pregnancy (hypertensive or diabetic disorders); gestational age at birth; mode of delivery; and offspring's sex. Height, BMI, and fat mass at age 17.8 years were considered as potential mediators if any confounder adjusted associations were observed. Offspring weight and height at the 17-year clinic assessment were measured using standard procedures in light clothing, without shoes and used to calculate BMI. Weight was measured to the nearest 0.1 kg using Tanita scales. Height was measured to the nearest 0.1 cm using a Harpenden stadiometer. A narrow fan beam densitometer (Lunar Prodigy; GE Healthcare Lunar Ltd) was used to perform a whole-body duel-energy X-ray absorptiometry scan from which fat mass was measured.

### Statistical analyses

Offspring liver volume, fibrosis, and liver function tests were log transformed to produce an approximately normal distribution of residuals. Birth weight was adjusted for sex and gestational age using nonlinear regression fitting a Gompertz curve.

Because human studies suggest that lower birth weight and animal studies suggest that higher birth weight is associated with a greater NAFLD risk, potential departure from linearity was explored by comparing a model in which the exposure was entered in thirds as two indicator variables and a model in which a linear relationship was assumed across the thirds, using a likelihood ratio test. The multivariable linear and logistic regression models were used to examine the associations between cord blood measures, birth weight and fatty liver, liver volume, fibrosis, and liver function tests at age 17 years. We tested for sex-exposure interactions, and because there was no evidence to support these, results are reported for male and female offspring combined.

A series of multivariable regression models were constructed to adjust for potential confounders and mediators. The basic model (model 1) adjusted for offspring sex and age at outcome assessment. In model 2 we additionally adjusted for potential confounders: maternal age, smoking in pregnancy, parity, occupational social class, education, prepregnancy BMI, and alcohol consumption in pregnancy. Our plan was to then examine potential mediation by adjusting for each of offspring BMI, fat mass, height, and height squared at age 17 years in any confounder-adjusted associations that were not null.

There were small amounts of missing data on some covariables included in the multivariable models. To deal with the missing data (any exposure, outcome or confounder), 20 imputation data sets were generated by chained equations (23), with measurements from the 15-year clinic informing imputation of missing values in the 17-year clinic, respectively. We imputed up to 541 (all participants with a liver scan and paired cord sample) and up to 1037 (participants with liver function tests [LFTs] and paired cord sample). The distributions of observed and imputed variables were similar (Supplemental Table 1). In the main paper, we present results from the imputed datasets and for comparison present results from those with complete data (n = 383–813) in the Supplemental Material (Supplemental Tables 2 and 3).

All analyses were performed using Stata MP version 12 (Stata Inc.).

## Results

Table 1 summarizes the maternal and offspring characteristics for those who attended the clinic at age 17 years and had liver function tests and/or a liver ultrasound scan. In most cases, the proportion of missing data for each variable was less than 5%. Baseline characteristics for the mothers and their young adult offspring were similar for those who had just the blood measures and those who had USS. Of the 541 who were assessed by ultrasound, only 10 (1.8%) were categorized as having a fatty liver.

**Table 1.** Characteristics of Those Who Had a Liver Scan (n = 541) and/or Liver Function Tests (n = 1037) Performed at Age 17 Years and Percentage With Missing Data

	Liver Scan			Liver Function Tests		
	Median or %, n Obs (IQR or n)	n Obs	Missing, %, n	Median or Obs, %, n, IQR, or n	n Obs	Missing, %, n
Maternal characteristics						
Age, y	29 (26, 32)	535	1.1 (6)	29 (26, 32)	1023	1.3 (14)
Smoking		525	3.0 (16)	77.1 (779)	1011	2.5 (26)
Never	74.9 (393)			7.6 (77)		
Before, not during, pregnancy	9.5 (50)			15.3 (155)		
During pregnancy	15.6 (82)					
BMI	22.3 (20.5, 24.4)	479	11.5 (62)	22.0 (20.5, 24.1)	918	11.5 (120)
Parity		508	6.1 (33)		991	4.4 (46)
0	46.7 (237)			47.1 (467)		
1	39.2 (199)			36.8 (365)		
2	9.7 (49)			11.8 (117)		
3	3.9 (20)			3.4 (34)		
4+	0.6 (3)			0.8 (8)		
Social class		452	16.5 (89)		884	14.6 (153)
I (least disadvantaged)	6.9 (31)			8.0 (71)		
II	38.5 (174)			38.4 (339)		
III	40.3 (182)			39.5 (349)		
IV	5.1 (23)			5.1 (45)		
V	8.4 (38)			7.6 (67)		
VI (most disadvantaged)	0.9 (4)			1.5 (13)		
Education		518	4.3 (23)		994	4.1 (43)
Left school at age 16 y	56.6 (293)			53.3 (530)		
A level	25.3 (131)			28.3 (281)		
Degree	18.1 (94)			18.4 (183)		
Alcohol		522	3.5 (19)		1009	2.7 (28)
None/<1 glass/wk	43.5 (227)			43.0 (434)		
>1 glass/wk	46.4 (242)			45.9 (463)		
>1 glass/d	10.2 (53)			11.1 (112)		
Offspring characteristics						
Sex		541	0.0 (0)		1037	0.0 (0)
Male	39.7 (215)			46.4 (481)		
Female	60.3 (326)			53.6 (556)		
Birth weight, kg	3.5 (3.2, 3.8)	530	2.0 (11)	3.5 (3.2, 3.8)	1013	2.3 (24)
Cord leptin, pg/mL	6.2 (3.7, 11.9)	539	0.4 (2)	6.3 (3.6, 11.9)	1035	0.2 (2)
Cord adiponectin, μg/mL	70.9 (51.0, 94.0)	533	1.5 (8)	73.5 (52.8, 95.0)	1026	1.1 (12)
Cord cholesterol, mmol/L	1.7 (1.5, 2.1)	529	2.2 (12)	1.7 (1.4, 2.0)	1021	1.6 (17)
Cord triglycerides, mmol/L	0.5 (0.4, 0.6)	524	3.1 (17)	0.5 (0.4, 0.6)	1016	2.1 (22)
Cord HDL-c, mmol/L	0.5 (0.4, 0.7)	518	4.3 (23)	0.5 (0.4, 0.7)	1005	3.1 (33)
Cord Non-HDL-c, mmol/L	1.2 (1.0, 1.5)	518	4.3 (2.3)	1.2 (0.9, 1.5)	1005	3.1 (33)
Liver volume, mL	1607.4 (1355.4, 1858.9)	541	0.0 (0)			
Liver velocity, m/sec	1.2 (1.1, 1.3)	500	7.9 (41)			
Diagnosis of fatty liver	1.8 (10)	371	31.4 (170)			
GGT, U/L			31.4 (170)	16 (13, 21)	1037	0.0 (0)
ALT, U/L			31.4 (170)	14.8 (12, 19.2)	1037	0.0 (0)
AST, U/L			4.8 (26)	19.3 (16.6, 23.3)	1004	3.1 (33)
Fat mass, kg	17.4 (11.8, 23.9)	522	3.6 (19)	16.3 (10.6, 23.0)	1011	2.5 (26)
BMI, kg/m^2^	22.2 (20.2, 24.8)	522	3.6 (19)	22.1 (20.2, 24.7)	1011	2.5 (26)
Height	169.8 (163.8, 177.8)			171.4 (164.5, 178.9)		
Diagnosis of obesity		31 (5.9)			51 (5.0)	

Abbreviations: IQR, interquartile range; obs, observations. Data are median (IQR) or percentage of number of observations (n observations).

There was no strong evidence of associations between birth weight, cord leptin, and lipids with ultrasound-diagnosed fatty liver (Table 2). Of 42 tests of associations of exposures with blood and ultrasound markers of liver health, two reached the conventional 5% significance level (Table 3). Birth weight was positively associated with liver volume (1.0% greater per 100 g [95% confidence interval [CI] 0.5, 2.0%]), and cord HDL-c was positively associated with ALT (11.6% higher per 1 mmol/L [95% CI 0.3, 23.4]). There was no evidence of associations between cord leptin or adiponectin with liver volume, velocity, or LFTs in adolescence. There was also no evidence of nonlinearity for birth weight or cord blood measures with any of the blood- or ultrasound-based liver outcomes.

**Table 2.** Association of Birth Weight and Cord Blood Measures With NAFLD in Adolescence (Using Multiple Imputation Data Sets) (n = 541)

Outcome Age 17 y Exposure	Diagnosis of Fatty Liver (Yes/No)		
	OR	CI	*P* Value
Cord leptin, per 10 pg/mL			
Model 1	0.68	0.26, 1.73	.41
Model 2	0.62	0.23, 1.65	.33
Cord adiponectin, per 10 μg/mL			
Model 1	0.85	0.70, 1.04	.13
Model 2	0.78	0.60, 1.01	.06
Cord cholesterol, per 1 mmol/L			
Model 1	0.81	0.36, 1.83	.62
Model 2	0.90	0.43, 1.87	.78
Cord triglycerides, per 1 mmol/L			
Model 1	0.74	0.09, 5.94	.78
Model 2	0.79	0.08, 8.20	.84
Cord HDL-c, per 1 mmol/L			
Model 1	0.51	0.04, 5.87	.58
Model 2	0.28	0.02, 3.78	.34
Non-HDL-c, per 1 mmol/L			
Model 1	0.82	0.33, 2.05	.67
Model 2	1.02	0.48, 2.15	.96
Birth weight, per 100 g^a^			
Model 1	1.00	0.88, 1.13	.98
Model 2	0.96	0.84, 1.10	.59

Adjustments for confounders included the following: model 1, offspring sex and age; and model 2, offspring sex and age, maternal age, smoking, parity, occupational social class, education, prepregnancy BMI, and alcohol consumption during pregnancy.

a Birth weight adjusted for gestational age and sex.

**Table 3.** Association of Birth Weight and Cord Blood Measures With Markers of Liver Health in Adolescence (Using Multiple Imputation Data Sets) (n = 541 for Liver Scans, n = 1037 for LFTs)

Outcome Age 17 y Exposure model (1 and 2)	LogLiver Volume, mL			LogLiver Sheer Velocity, m/sec			LogALT, U/L			LogAST, U/L			LogGGT, U/L		
	%	CI	*P* Value	%	CI	*P* Value	%	CI	*P* Value	%	CI	*P* Value	%	CI	*P* Value
Cord leptin, per 10 pg/mL															
Model 1	1.0	−1.0, 3.0	.22	−0.03	−2.0, 1.0	.97	0.4	−2.0, 3.0	.73	0.2	−1.0, 2.0	.81	−0.1	−2.0, 2.0	.96
Model 2	0.3	−2.0, 2.0	.79	−1.0	−2.0, 1.0	.46	−0.6	−3.0, 2.0	.61	−0.03	−2.0, 2.0	.97	−0.5	−3.0, 2.0	.66
Cord adiponectin, per 10 μg/mL															
Model 1	−0.2	−1.0, 1.0	.61	0.1	−1.0, 1.0	.82	−0.2	−1.0, 1.0	.68	0.3	−0.2, 1.0	.23	0.2	−0.4, 1.0	.41
Model 2	−0.1	−1.0, 1.0	.79	0.04	−0.6, 0. 6	.99	−0.3	−1.0, 1.0	.53	0.3	−0.2, 0.8	.27	0.3	−0.4, 0.9	.45
Cord cholesterol, per 1 mmol/L															
Model 1	0.1	−2.0, 2.0	.90	−1.0	−3.0, 1.0	.24	1.0	−1.0, 4.1	.22	−0.1	−2.0, 1.0	.85	−0.1	−2.0, 2.0	.90
Model 2	0.02	−2.0, 2.0	.98	−1.0	3.0, 1.0	.38	1.0	−1.0, 4.1	.25	−0.2	−2.0, 1.0	.82	−0.03	−2.0, 2.0	.97
Cord triglycerides, per 1 mmol/L															
Model 1	0.4	−5.8, 7.3	.91	−2.0	−7.7, 3.0	.45	2.0	−3.9, 8.3	.53	−0.01	−4.9, 4.1	.76	−2.0	−6.8, 3.0	.49
Model 2	−1.0	−7.7, 5.8	.73	−2.0	−7.7, 3.0	.41	1.0	−4.9, 8.3	.67	−0.01	−4.9, 3.0	.71	−2.0	−1.0, 4.1	.32
Cord HDL-c, per 1 mmol/L															
Model 1	1.0	−7.7, 9.4	.88	2.0	−4.9, 9.4	.57	10.5	−1.0, 22.1	.07	3.0	−3.9, 10.5	.42	2.0	−6.8, 11.6	.63
Model 2	2.0	−5.8, 11.6	.60	4.0	−3.0, 11.6	.22	11.6	0.3, 23.4	.04	3.0	−3.9, 10.5	.40	3.0	−5.8, 12.7	.47
Non-HDL-c, per 1 mmol/L															
Model 1	−0.1	−2.0, 2.0	.97	−2.0	−3.0, 0.0	.13	1.0	−1.0, 4.1	.36	−0.4	−2.0, 1.0	.69	−0.3	−3.0, 2.0	.81
Model 2	−0.2	−3.0, 2.0	.88	−1.0	−3.0, 0.6	.18	1.0	−2.0, 4.1	.43	−0.4	−2.0, 2.0	.64	−0.2	−2.0, 2.0	.83
Birth weight, per 100 g^a^															
Model 1	1.0	1.0, 2.0	.000	0.3	−0.1, 1.0	.12	−0.4	−1.0, 0.2	.22	−0.3	−1.0, 0.1	.12	−0.2	−1.0, 0.3	.42
Model 2	1.0	0.3, 1.0	.001	0.1	−0.3, 0.5	.69	−1.0	−1.0, 0.02	.06	−0.4	−1.0, 0.02	.06	−0.4	−1.0, 0.1	.15

Adjustments for confounders included the following: model 1, offspring sex and age; and model 2, offspring sex and age, maternal age, smoking, parity, occupational social class, education, prepregnancy BMI, and alcohol consumption during pregnancy.

a Birth weight adjusted for gestational age and sex.

To examine the effects of potential mediators, we included offspring fat mass, height (and height squared), and BMI at age 17 years in the fully adjusted models. The association between birth weight and increased liver volume remained (1.0% greater per 100 g, 95% CI 0.1%, 1.0%), whereas the association of cord HDL-c and ALT changed direction (1.0% lower per 1 mmol/L, 95% CI −2.0, −0.4).

Associations were similar when restricted to a complete case, ie, including participants who had data on all off the exposures, confounders, and outcomes (Supplemental Tables 2 and 3).

## Discussion

In this study, we assessed prospective associations of birth weight and cord blood measures of leptin, adiponectin, and lipids with markers of adolescent liver structure and function. Of a total of 42 confounder-adjusted tests, we identified two that were significant at conventional 5% levels: a positive association of birth weight with adolescent liver volume and cord HDL-c with adolescent ALT. Overall, these results do not suggest that birth weight, birth fat (as assessed by cord leptin and adiponectin) or cord lipids are associated with later offspring markers of liver health when adjustment is made for maternal pregnancy characteristics that are likely to confound these associations.

Birth weight has been associated both positively and negatively with liver transaminases (5, 18, 24, 25) and a greater risk of NAFLD, liver cirrhosis, and mortality (4, 24, 26). These nonconsistent findings may reflect the problems with using birth weight, which is a crude marker of the sum of antenatal and intrauterine exposures and also fat and lean mass. We tried to extend these studies by using cord blood adiponectin and leptin as surrogates of neonatal fat mass.

Adiponectin has consistently shown to be reduced in children and adults with confirmed NAFLD (27–30). Conversely, in an earlier publication using the same cohort as used here, strong positive associations of leptin levels (as well as fat mass and BMI) at age 17 years with liver fat were reported. Serum lipids are also known exhibit strong positive associations with adverse liver function in cross-sectional analyses (30, 31). That we did not observe a prospective longitudinal association of cord blood adiponectin or leptin with markers of liver function in adolescence suggests that their contribution at birth to the pathogenesis of childhood NAFLD is limited. That cord HDL-c was positively associated with ALT at age 17 years may have been a chance finding. Collectively this suggests that although leptin and adiponectin reflect neonatal mass, disease in adolescence is primarily driven by childhood behavior rather than intrauterine exposures. Consistent with this, cross-suckling experimental data suggest that exposure to maternal obesity only via lactation more aggressively programs a dysmetabolic and NAFLD phenotype than intrauterine exposure (8).

Whereas previous studies of birth weight with later liver outcomes have been larger than this study, to our knowledge this is the largest study to examine cord blood measures in relation to subsequent liver outcomes, including measurements assessed by USS. The prospective design, long duration of follow-up, detailed anthropometric measures, and appropriate adjustment for maternal characteristics that could confound the associations are substantive strengths. We do, however, acknowledge several limitations. We used ultrasound to assess liver pathology because it has been shown to detect moderate to severe steatosis with similar accuracy as the gold standard liver biopsy while avoiding an unethical and invasive procedure (21). For lesser degrees of hepatic fat mass, however, ultrasound may not be as sensitive a tool. The absence of associations in this study may reflect this, particularly because a young adolescent population is more likely to exhibit subtle changes in hepatic structure because the disease process itself may still be in its infancy. Further assessment in later life may provide additional information, by allowing time for liver abnormalities to fully manifest. Analyses were limited to those with an available cord blood sample and to those participants who attended the 17-year-old clinic; however, although we cannot directly test this, it is unlikely that this should bias results. Multiple imputation methods were used to account for missing confounder data, with a similar finding in those with complete data on all confounders. The ALSPAC cohort is mainly of white European origin, and as a result, this study may not reflect other ethnicities that have previously been strongly linked with hepatic dysfunction and elevated ALT levels (32). Lastly, we acknowledge that multiple comparisons were performed and our positive findings may have been chance findings.

In animal models, insulin resistance, hyperketonemia, and hepatic steatosis were all features of male but not female offspring of obese mice (33), and there have been inconsistent reports of gender-specific predispositions to NAFLD. In the current study, most offspring participating in this study were female (60.3%), and this may account for the low numbers with evidence of NAFLD. Examining offspring outcomes in adolescence may be too early in life to identify those who go on to develop metabolic disorders such as NAFLD and type 2 diabetes. Our findings may therefore underestimate the true prevalence of the disease and account for the lack of associations in this study. Cord blood sample degradation may have contributed to variability within the results, but leptin, adiponectin, and lipids do appear to be stable with long-term storage (34–37).

In the current study, there was no consistent evidence that birth weight, neonatal adiposity, or cord lipids were associated with liver dysfunction in adolescence. The association of maternal adiposity to later offspring NAFLD is likely to be a consequence of later environmental effects or in utero effects not mediated via in utero adiposity.
